# Assessment of MDM2 Gene Locus Amplification by Fluorescence In-Situ Hybridization in Juvenile Ossifying Fibroma

**DOI:** 10.1007/s12105-024-01682-x

**Published:** 2024-08-06

**Authors:** Faraj Alotaiby, Saja A. Alramadhan, Sarah G. Fitzpatrick, Mohammed N. Islam, Donald M. Cohen, Indraneel Bhattacharyya

**Affiliations:** 1 Department of Oral and Maxillofacial Diagnostic Sciences, College of Dentistry, Qassim University, Buraydah, Saudi Arabia; 2 Department of Oral and Maxillofacial Surgery and Pathology, University of Mississippi Medical Center, Jackson, MS USA; 3 Department of Oral and Maxillofacial Diagnostic Sciences, University of Florida College of Dentistry, Gainesville, FL USA

**Keywords:** Benign fibro-osseous, Juvenile Ossifying Fibroma, MDM2, Osteosarcoma, Low-grade Intramedullary Osteosarcoma, FISH

## Abstract

**Abstract:**

Juvenile ossifying fibroma (JOF) is an uncommon benign fibro-osseous lesion (BFOL) of the maxillofacial bones with a locally aggressive nature and a high recurrence rate. Murine Double Minute 2 (MDM2) is an oncogene located at chromosome 12 (12q13-15) that inhibits the tumor suppressor gene TP53. The presence of MDM2 gene locus amplification is a useful molecular adjunct in the evaluation of some sarcomas, including low-grade intramedullary osteosarcoma (LGIOS). JOF and LGIOS have some overlapping clinical and histopathological features. The aim of this study is to evaluate a series of JOF for the presence of MDM2 gene locus amplification using fluorescence in-situ hybridization (FISH).

**Materials and methods:**

With IRB approval, a search of the institutional files of the archives of the Oral Pathology and Surgical Pathology biopsy services at the University of Florida Health was performed. The cases were re-evaluated by an oral pathology resident, an oral and maxillofacial pathologist, and a bone and soft tissue pathologist. Cases with consensus in diagnosis were selected (*n* = 9) for MDM2 testing. Testing by FISH for MDM2 gene locus amplification was applied to all retrieved cases.

**Results:**

The examined cases were all negative for MDM2 gene locus amplification via FISH testing.

**Conclusion:**

In our small series, JOF did not demonstrate MDM2 gene locus abnormality, a characteristic of LGIOS. This finding suggests that JOF has a distinct underlying pathogenesis. If confirmed in a larger series, these findings may be useful in distinguishing these two entities in cases with overlapping features or when minimal biopsy material is available.

## Introduction

Juvenile ossifying fibroma (JOF) is an uncommon benign fibro-osseous lesion of the maxillofacial bones with locally aggressive behavior and a high recurrence potential [1–4]. It is divided into two subtypes: trabecular and psammomatoid, each with distinct clinical and histopathologic features [1, 2].

Trabecular juvenile ossifying fibroma (TJOF) affects young patients with an age range of 8.5–12 years without significant gender predilection [1–3]. Gnathic bones are the most affected sites, with remarkable maxillary involvement in comparison to the mandible [1, 2]. Rapid and progressive expansion is the classic clinical presentation. Nasal obstruction and epistaxis are seen with maxillary lesions [1–4]. Radiographs typically reveal expansile, well-demarcated lesions causing thinning or perforation of cortical bone. Tumors have a mixed radiolucent/opaque appearance which can vary depending on the amount of calcified tissue present [1, 2]. Microscopically, TJOF is usually unencapsulated and infiltrates the surrounding bone. The stroma is hypercellular and composed predominantly of spindle or polyhedral cells with minimal collagen production [1–4]. Cellular, immature osteoid forms strands that can be either long and slender or plump. The immature osteoid is sometimes hard to distinguish from the cellular stroma. Immature bone trabeculae surrounded by osteoblastic rimming are typically present, but maturation to lamellar bone does not occur. Local aggregates of osteoclastic giant cells are consistently found in the stroma. Mitotic activity in the stromal cells can be present, raising the possibility of osteosarcoma (OS), but it is never numerous. In some cases, cystic degeneration and aneurysmal bone cyst formation have been reported. [1–5].

Psammomatoid juvenile ossifying fibroma (PJOF) affects mostly the extragnathic craniofacial bones, with periorbital, ethmoid, and frontal bones being the most affected sites [1, 3–5]. Patients with PJOF are older compared to the trabecular variant with a wider age range between 3 months to 72 years. Most reported cases are between the ages of 16–33 [1, 3, 4]. As is the case with TJOF, no gender predilection has been reported [1, 3, 4]. PJOF presents as a bony enlargement that may involve the orbit, nasal bone, and sinuses causing proptosis, visual symptoms, and nasal obstruction [1–5]. Radiographic examination usually demonstrates a well-demarcated radiolucent lesion with a ground-glass pattern, which varies in size from 2 to 8 cm in diameter [2, 5]. Histologically, the tumor is unencapsulated and composed of cellular fibrous connective tissue containing numerous small and uniform ossicles known as psammomatoid bodies. The psammomatoid bodies are basophilic, resembling cementum, and may coalesce at the periphery of the lesion to form bony trabeculae [2, 4, 5]. Evidence of cystic degeneration and aneurysmal bone cyst formation may also be present [2, 6].

Gnathic benign fibro-osseous lesions, including JOF, have some overlapping clinical, radiological, and histomorphologic features with low-grade intramedullary osteosarcoma (LGIOS) [2, 6]. Although both tumors have high recurrence rates, LGIOSs are malignant tumors that carry a 15–29% risk of de-differentiation, particularly in the case of recurrent tumors [7–9]. LGIOSs usually do not demonstrate the typical microscopic features of malignancy [8]. They exhibit moderately cellular fascicles of spindle cells with mild nuclear atypia and neoplastic woven or lamellar bone [8]. Radiographically, gnathic LGIOSs may appear as either speckled radiopaque, mixed radiolucent-radiopaque, or radiolucent lesions with ragged, poorly defined borders [7]. A classic sunray, sunburst, moth-eaten pattern or Codman triangle may also be evident [7, 9]. The tumor tends to invade and damage adjacent anatomical structures. The involved teeth may show signs of spiking root resorption and symmetrical widening of the periodontal ligament space [7]. However, these features are not always seen.

Tumorigenesis often results from disrupted regulation of the cell cycle and apoptosis. Research indicates that the p53-MDM2 and Rb-Cyclin D-CDK4 pathways, which control the G1-S phase transition of the cell cycle, are implicated in the development of OS, including its gnathic variants. Murine Double Minute 2 (MDM2) is an oncogene located at chromosome 12 (12q13-15) which inhibits the tumor suppressor gene TP53 [10]. P53 is activated as a result of certain cellular stresses [10]. This results in apoptosis, cell cycle arrest, DNA repair, and senescence, which preclude tumor initiation and progression [10]. MDM2 is considered a negative regulator of p53. High levels of MDM2 expression reduce p53 protein levels and function, which in turn accelerates cancer development and progression [11]. MDM2 gene locus amplification is a useful molecular finding in the diagnosis of some sarcomas, including LGIOS, parosteal osteosarcoma, and liposarcoma [12]. MDM2 amplification has been studied as a predictive biomarker for tumors’ behavior, prognosis, and therapeutic target [12]. The cyclin Dependent Kinase 4 (CDK4) gene is located at chromosome 12q14.1, assists in the G1 to S phase cell cycle transition. CDK4 is usually amplified in tumors that have MDM2 amplification [12]. While the role of these genes in malignant bone tumors is well-documented, their involvement in benign jaw tumors like JOF is less clear.

Different methods are being used to detect MDM2 amplification such as immunohistochemistry, polymerase chain reaction, comparative genomic hybridization, and fluorescent in situ hybridization (FISH) [11–13]. However, the latter is a valuable diagnostic tool to detect MDM2 amplification in the clinical setting [12, 13]. The FISH technique can determine the MDM2 copy number gain and MDM2 ratio in reference to a standard centromeric probe (CEP12) [12]. The aim of this study is to evaluate a series of JOF for the presence of MDM2 gene locus amplification using FISH.

## Materials and Methods

With IRB approval, institutional archives for the University of Florida (UF) Oral Pathology biopsy service and UF Health Anatomic Pathology service were queried for diagnosed JOF cases over 20 years, from 1999 to 2018. All cases were reviewed by a senior oral pathology resident, an oral pathologist, and a bone and soft tissue pathologist. Cases with insufficient material for testing, or material unsuitable for testing, such as specimens that were previously decalcified, were excluded. In addition, cases with uncertain diagnoses were also excluded. Testing for MDM2 gene locus amplification by fluorescent in situ hybridization (FISH) was performed on formalin-fixed, paraffin-embedded (FFPE) tissue.

### FISH Interphase Study

Nine paraffin-embedded archival tissue samples were chosen for this study: seven were of the psammomatoid variant and two were of the trabecular variant. Each sample was sectioned at 4 mm. FISH was utilized to assess these cases, targeting the chromosomal regions MDM2 (12q15) and D12Z3 (Centromere 12) with dual-color break-apart probe. The MDM2 region probe was labeled in orange, while the Centromere 12 region probe was labeled in green. Following hybridization, slides were counterstained with 4′,6-diamidino-2-phenylindole (DAPI).

A minimum of 40 nuclei were counted per case. Nuclei with at least two 12cen probe signals were included to minimize potential nuclear truncation artifacts. Overlapping nuclei were excluded to avoid false positive results. An aberrant hybridization signal pattern, indicating MDM2 probe signal amplification, was defined as an MDM2/12cen ratio greater than 2.0. A ratio below 2.0 was considered non-amplified. The normal cut-off value for this aberrant hybridization signal pattern was determined using concurrently hybridized MDM2 and D12Z3 DNA probes in negative FFPE control samples, yielding 1.57% (or 5/300 positive observations at a 95% confidence level).

## Results

**Fig. 1:**
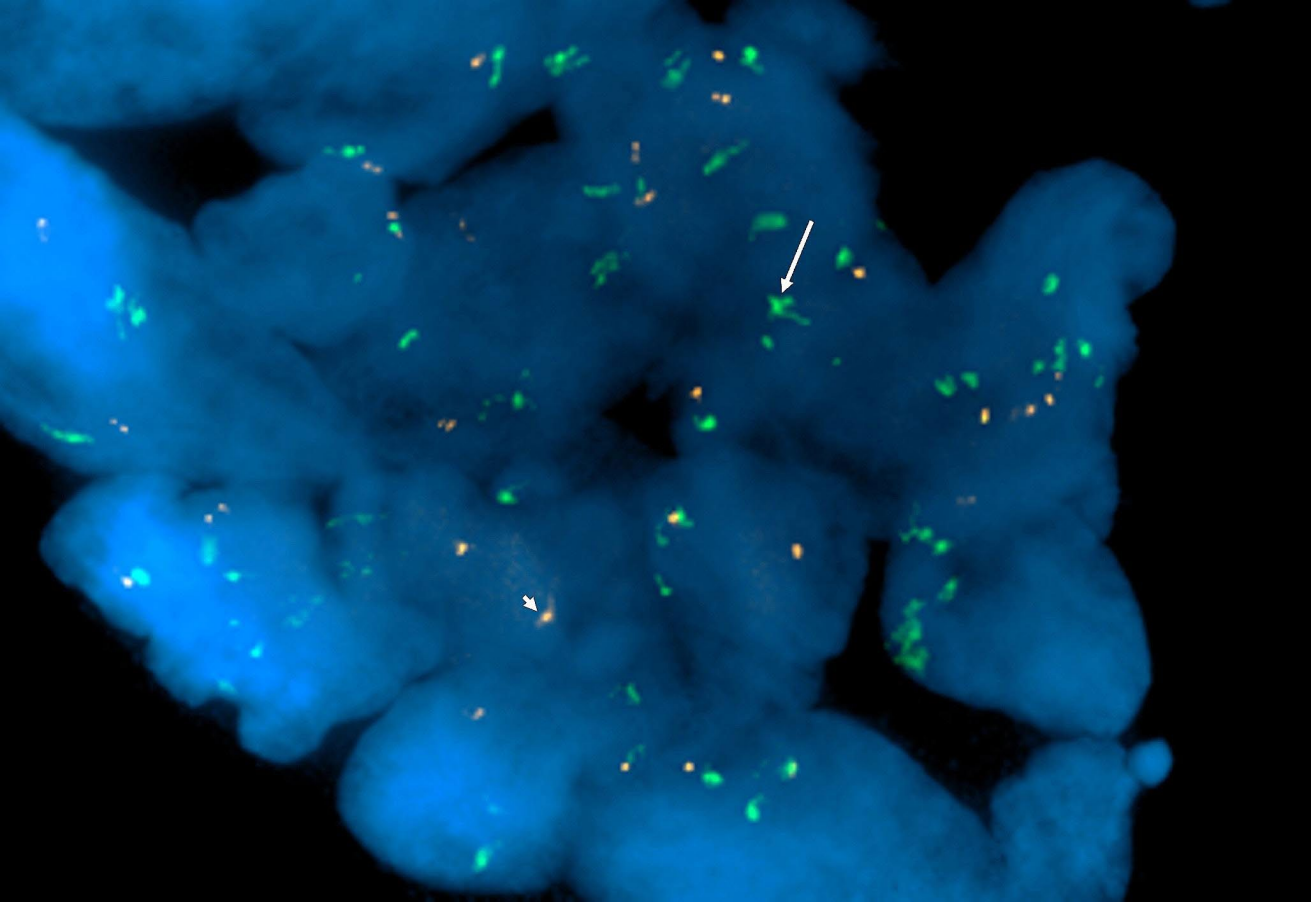
Florescent in-situ hybridization for case 5 of juvenile ossifying fibroma. The green probe signal labels the centromere (arrow). The probe labels the MDM2 (arrowhead). Abnormal pattern of 12 centromeric probe signals copy exceeds MDM2 number

An underlying chromosome 12q15 rearrangement resulting in an amplification of the MDM2 gene locus was not detected in any of the cases by interphase FISH methods. A single case, Case 5, showed an abnormal pattern where the 12 centromeric probe signals exceeded the number of MDM2 probe signals, but no MDM2 amplification was noted (Fig. 1). Features of the cases, including demographics, clinical features, histologic subtypes, and results of FISH testing, are summarized in Table 1.

**Table 1:** Clinical, histologic features and MDM2 results of examined juvenile ossifying fibroma cases

Case	Location	Gender	Age	Histologic Variant	MDM2 Amplification by FISH	Copies of CEP12 and MDM2 Signals	Comments
1	Maxilla	F	21	Psammamatoid	Not detected	< 2	
2	Ethmoid	F	12	Psammamatoid	Not detected	< 2	
3	Maxilla and nasal	F	18	Psammamatoid	Not detected	< 2	
4	Mandible	M	12	Psammamatoid	Not detected	< 2	
5	Maxilla and nasal	M	26	Trabecular	Not detected	~ 3:1	Frequency of 12cen probe signals exceeded that of the MDM2 number
6	Maxilla	F	29	Psammamatoid	Not detected	< 2	
7	Mandible	F	24	Psammamatoid	Not detected	< 2	
8	Mandible	F	17	Trabecular	Not detected	< 2	
9	Maxilla	F	21	Psammamatoid	Not detected	< 2	

## Discussion

According to the 5th edition of the World Health Organization’s (WHO) Classification of Head and Neck Tumors, juvenile ossifying fibroma (JOF) is a specific type of ossifying fibroma (OF), notable for its rapid growth, local invasiveness, and tendency to recur. Diagnosing JOF requires a comprehensive clinicopathologic approach, which includes evaluating the patient’s age at onset, tumor location, clinical and radiologic features, microscopic characteristics, and biological behavior. Two distinct subtypes of JOF are recognized: trabecular juvenile ossifying fibroma (TJOF) and psammomatoid juvenile ossifying fibroma (PJOF).

**Fig. 2:**
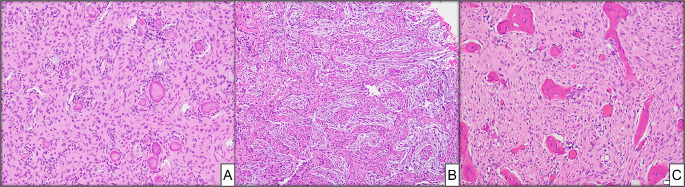
Histological comparison of juvenile ossifying fibroma (JOF) and low-grade intramedullary osteosarcoma shows irregular bone trabeculae within variably cellular stroma. **(A)** Psammomatoid variant JOF, magnification X100 (H&E*). **(B)** Trabecular variant JOF, magnification X100 (H&E*). **(C)** Low grade osteosarcoma (comparison case from University of Florida Department of Pathology, Immunology, and Laboratory Medicine Archives), magnification X200 (H&E*). *Hematoxylin-eosin staining

The main differential diagnoses include other fibro-osseous lesions and malignant tumors such as intramedullary low-grade osteosarcoma (LGIOS). Although specific clinicopathologic traits generally allow for differentiation, some borderline cases remain diagnostically challenging. The distinction between LGIOS and JOF can be difficult due to their overlapping clinical and histopathologic features (Fig. 2). Both tumors can have dense stroma, mitotic figures, infiltrative growth, and immature and woven calcifications, which appear to develop directly from stromal cells [13]. The bland cytological and nuclear features of low-grade osteosarcoma can lead to misdiagnosis [7, 9, 13]. Initial misdiagnosis of low-grade osteosarcomas as benign tumors was reported in 32–60% of cases in two literature reviews [14, 15]. Another study conducted in a specialized hospital demonstrated misdiagnosis in half of the low-grade osteosarcoma cases [15]. A reliable laboratory test that differentiates JOF from low-grade osteosarcoma would help resolve this significant issue.

Cytogenetic studies of a few cases of JOF have been published [16]. One study demonstrated that all cases of PJOF of the orbit had nonrandom chromosome breakpoints at Xq26 and 2q33, resulting in (X:2) translocations [16]. Tabareau-Delalande et al. analyzed MDM2 amplification using qPCR in benign fibro-osseous lesions of the jaws, including JOF, and identified MDM2 amplification in 69% of JOF cases [17]. This finding, if confirmed, could certainly have implications for the classification of JOF. However, other benign fibro-osseous lesions in their study also showed MDM2 gene locus amplification, including 12% of fibrous dysplasias [17]. In contrast, the present study found that none of the cases demonstrated MDM2 gene locus amplification by FISH.

Dujardin et al. compared the immunohistochemical expression of MDM2 and CDK4 in low-grade osteosarcoma and benign fibro-osseous lesions [18]. They found that all benign fibro-osseous lesions were negative for MDM2 and CDK4, while 89% of low-grade osteosarcomas were positive for both tests [18]. In addition, Kansara et al. concluded that MDM2 amplification using FISH can differentiate low-grade osteosarcoma from clinically and microscopically similar benign fibro-osseous lesions [19]. Our study aligns with these findings.

However, Nikitakis et al. reported that three out of five JOF cases showed mild to moderate MDM2 immunohistochemical positivity, suggesting that MDM2 aberrations may contribute to its pathogenesis and aggressive behavior [20]. These conflicting results highlight the need for further research to clarify the role of MDM2 in JOF. Understanding the molecular pathways involved in JOF could help identify markers to distinguish it from other benign and malignant bone lesions.

## Conclusion

JOF did not demonstrate MDM2 gene locus amplification, a characteristic of LGIOS, indicating the likelihood of distinctly separate pathogenesis despite some overlapping morphologic features. These findings may be a useful adjunct in differentiating between these two entities, especially in cases with microscopic overlap or in small biopsy specimens. Utilizing both FISH and qPCR techniques concurrently in a single study to evaluate MDM2 gene locus amplification may be helpful in future studies to differentiate these lesions. Additionally, studies with larger sample sizes are needed to validate the diagnostic utility of this marker and to investigate its prognostic and predictive significance.

## Data Availability

No datasets were generated or analysed during the current study.
